# Spontaneous rupture of muscular hydatid cyst

**DOI:** 10.1590/0037-8682-0363-2023

**Published:** 2023-09-22

**Authors:** Recep Tekin, Emin Özkul, Rojbin Ceylan Tekin

**Affiliations:** 1 Dicle University, Faculty of Medicine, Department of Infectious Diseases and Clinical Microbiology, Diyarbakir, Turkey.; 2 Dicle University, Faculty of Medicine, Department of Trauma and Orthopedic Surgery, Diyarbakir, Turkey.; 3 Dagkapi State Hospital, Department of Radiology, Diyarbakir, Turkey.

**FIGURE 1:**
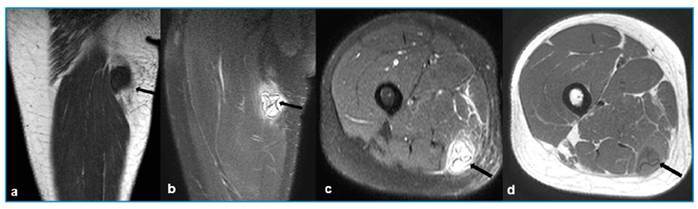
**a-b,** MRI of the patient’s right thigh revealed T1-weighted hypointense and T2-weighted fat-suppressed series hyperintense lesions in the semimembranosus muscle (arrow). **c-d**, MRI thigh shows rupture of a muscular hydatid cyst with a “water lily sign” produced by detachment of the germinal membrane of the endocyst (arrow).

**FIGURE 2:**
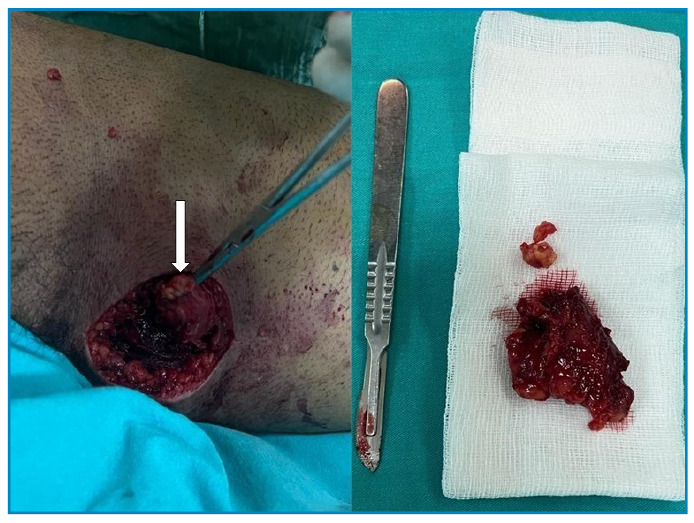
Image from an intraoperative procedure.

**FIGURE 3:**
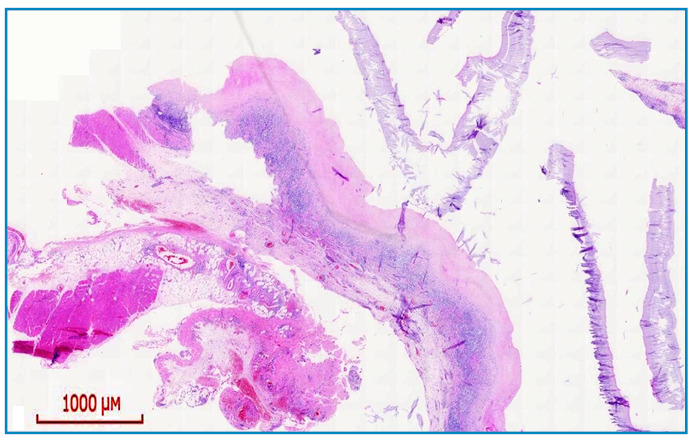
Depiction of the cyst wall, including the inner germinal layer and laminated membrane (H&E stain; x40 magnification). **H&E:** Hematoxylin and Eosin.

A 23-year-old male patient reported experiencing pain in his right thigh for 2 days along with swelling in the same area for the past 5 months. Upon physical examination, his thigh was found to be mildly painful. Magnetic resonance imaging (MRI) revealed T1-weighted hypointense and T2-weighted fat-suppressed series hyperintense lesions in the semimembranosus muscle (Figure 1a,b). The MRI further indicated a rupture of a muscular hydatid cyst, as evidenced by the “water lily sign” produced by the detachment of the germinal membrane of the endocyst (Figure 1c,d arrow). The MRI also confirmed the intramuscular location of the lesion in the semimembranosus muscle, which exhibited significant edematous signal changes in the surrounding muscle structures and between the fascia planes. Based on the clinical, laboratory, and radiological findings, the patient was diagnosed with a ruptured muscular hydatid cyst. A wide resection of the right thigh was performed, ensuring adequate margins of healthy tissue were maintained around the associated soft-tissue cystic components (Figure 2). The diagnosis of hydatid disease was confirmed through a histopathological examination (Figure 3). The patient was prescribed 400 mg of oral albendazole daily. The spontaneous rupture of muscular hydatid cysts is an extremely rare condition. The rupture of hydatid cysts into the muscle poses a considerable challenge for surgeons. Pericystectomy is the primary treatment for the muscular rupture of hydatid cysts, with medical treatment typically administered postoperatively^1^ ^,^ ^2^. It is important to consider hydatid disease in the differential diagnosis of cystic swelling in a musculoskeletal region^3^.
